# Acidic pH Modulates Cell Wall and Melanization in *Paracoccidioides brasiliensis*, Affecting Macrophage Interaction

**DOI:** 10.3390/jof11070504

**Published:** 2025-07-04

**Authors:** Rafael de Souza Silva, Wilson Dias Segura, Rogéria Cristina Zauli, Reinaldo Souza Oliveira, Vitor Vidal, Eduardo Correa Louvandini, Patricia Xander, Suzan Pantaroto Vasconcellos, Wagner Luiz Batista

**Affiliations:** 1 Departamento Microbiologia, Imunologia e Parasitologia, Escola Paulista de Medicina, Universidade Federal de São Paulo, São Paulo 04023-062, SP, Brazil; rs.silva02@unifesp.br (R.d.S.S.); wilson.dias@unifesp.br (W.D.S.); 2 Departamento de Ciências Farmacêuticas, Instituto de Ciências Ambientais, Químicas e Farmacêuticas, Universidade Federal de São Paulo, Diadema 09913-030, SP, Brazil; rczauli@unifesp.br (R.C.Z.); reinaldo.souza@unifesp.br (R.S.O.); vitor.vidal@unifesp.br (V.V.); eduardo.louvandini@unifesp.br (E.C.L.); patricia.xander@unifesp.br (P.X.); suzan.pantaroto@unifesp.br (S.P.V.)

**Keywords:** *Paracoccidioides brasiliensis*, melanin, acid pH, cell wall, macrophage

## Abstract

*Paracoccidioides brasiliensis* is a thermally dimorphic fungal pathogen and the main etiological agent of paracoccidioidomycosis (PCM), a neglected systemic mycosis endemic in Latin America. The virulence of *P. brasiliensis* is closely associated with its capacity to survive under hostile host conditions, including acidic environments. In this study, we demonstrate that acidic pH induces melanization in *P. brasiliensis*, modulates its cell wall composition, and alters the interaction with macrophages. Cultivation at acidic pH resulted in reduced fungal growth without compromising viability and triggered increased production of melanin-like pigments, as confirmed by enhanced laccase activity and upregulation of genes in the *DHN*-melanin biosynthetic pathway. Additionally, growth under acidic pH induced significant remodeling of the fungal cell wall, leading to increased chitin on the cell wall surface and reduced mannan content, while β-glucan levels remained unchanged. These modifications correlated with decreased viability to Congo Red, suggesting altered cell wall stability. Importantly, *P. brasiliensis* grown under acidic conditions exhibited reduced phagocytosis by RAW 264.7 macrophages, along with changes in nitric oxide and cytokine production, indicating potential mechanisms of immune evasion. Collectively, our findings suggest that environmental acidification promotes fungal adaptations that enhance survival and modulate host–pathogen interactions, contributing to *P. brasiliensis* virulence. Understanding how acidic pH regulates these processes provides new insights into the pathobiology of PCM and may contribute to understanding the mechanisms of fungal immune evasion.

## 1. Introduction

*Paracoccidioides* spp. are thermally dimorphic fungi responsible for paracoccidioidomycosis (PCM), a systemic mycosis that is a significant public health concern in Latin America. The disease is primarily transmitted through the inhalation of fungal conidia, which convert into yeast forms in the host’s lungs due to the body’s temperature [1]. *P. brasiliensis* is a fungal species within the family Ajellomycetaceae, order Onygenales. Additionally, part of this family includes the fungi *Histoplasma capsulatum*, *Blastomyces dermatitidis*, and *Coccidioides immitis.* The *Paracoccidioides* genus comprises multiple cryptic species that are responsible for PCM. Molecular studies have revealed distinct phylogenetic lineages within the species complex [1]. PCM often begins with pulmonary symptoms and can spread to other organs if untreated, leading to chronic conditions. This mycosis disproportionately affects rural populations engaged in agricultural activities due to the fungus’s environmental reservoir in soil and vegetation, where individuals are exposed to airborne conidia [2,3].

Epidemiological data show that PCM is endemic in Brazil, Argentina, Colombia, and Venezuela, with Brazil accounting for more than 80% of reported cases. The disease predominantly affects men aged 30–60, likely due to occupational exposure and the protective role of estrogen against fungal proliferation in women [4,5]. The incidence is estimated to be 1–3 cases per 100,000 individuals in endemic regions, but underreporting remains a challenge. Advances in diagnostic techniques and epidemiological surveillance are important for better management and control of PCM, considering its high morbidity and socioeconomic impact in affected areas [2,6].

*P. brasiliensis* virulence is closely linked to its ability to adapt and evade host immune responses. Crucial factors contributing to its pathogenicity include the ability to undergo thermal dimorphism, to modulate cell wall composition, and to produce melanin. Melanization in *P. brasiliensis* is a crucial defense mechanism that enhances the fungus’s survival by shielding it from oxidative stress, a significant component of the host’s immune response. Melanin can scavenge reactive oxygen species (ROS) and reactive nitrogen species (RNS) produced by phagocytic cells, such as macrophages, thereby reducing fungal susceptibility to oxidative damage [7].

Additionally, melanin in *P. brasiliensis* contributes to immune evasion by masking antigenic surface components, thus impairing recognition and phagocytosis by immune cells [7]. This pigment also influences the pathogen’s interaction with the host by modulating cytokine production, which can reduce pro-inflammatory responses and facilitate the persistence of infection [8]. Thus, understanding the role of melanin and other virulence factors in the host–pathogen interaction is critical for developing new therapeutic strategies against PCM [9].

The acidic pH is a significant environmental factor that influences the survival of pathogenic fungi, including *P. brasiliensis*. Our group has conducted systematic studies on the ability of *P. brasiliensis* to survive under different types of stress. Among the most relevant findings, we performed proteomic analyses on the Pb18 isolate with varying degrees of virulence (virulent Pb18 and attenuated Pb18). These studies revealed that proteins differentially expressed in the virulent isolate have the potential to act as virulence factors [10]. Additionally, we characterized the acidic proteases of *P. brasiliensis*, identifying four aspartyl proteases with high homology to the aspartyl protease Pep4 from *Saccharomyces cerevisiae*. We observed that culturing the fungus at pH 4.0 increases the secretion of these enzymes, which demonstrated the ability to cleave proteins such as bovine serum albumin (BSA), collagen, and hemoglobin [11,12]. These adaptations contribute to the fungus’s survival and interfere with the immune system’s ability to eliminate the pathogen by modulating the interaction of the fungus with phagocytic cells [13]. Additionally, the response to acidic pH is often associated with the regulation of signaling pathways that affect the expression of genes related to virulence and metabolism, thereby enhancing the fungus’s ability to persist in hostile environments [14]. Therefore, the characterization of *P. brasiliensis* responses to acidic pH provides relevant insights into potential adaptive traits that may contribute to its survival in the host environment. This study aimed to investigate how *P. brasiliensis* responds to acidic pH conditions. We demonstrate that acidic pH induces melanization, modulates cell wall composition, and alters the interaction of *P. brasiliensis* with macrophages. By exploring the physiological and molecular responses of the fungus under these conditions, we aim to deepen our understanding of the adaptive mechanisms that contribute to its virulence and ability to persist within the host.

## 2. Materials and Methods

### 2.1. Fungal Isolate and Growth Conditions

*P. brasiliensis* (Pb18 isolate) was grown in a yeast peptone dextrose-modified medium (mYPD) (0.5% yeast extract, 1% peptone, and 0.5% glucose, pH 6.5) for 4 to 5 days at 37 °C and shaking at 110–150 rpm. For mycelium growth, viable yeast cells were cultivated in mYPD or F-12 medium (supplemented with glucose 2%) (Gibco^®^, Waltham, MA, USA) at 25 °C for 7 days at 110–150 rpm. Viability was assessed using Trypan blue 0.4% counting on Neubauer’s chamber. To ensure the recovery of fungal virulence, it is a routine procedure in our laboratory to infect BalbC mice and subsequently isolate yeast cells from infected tissues, as described by Castilho et al. [10]. This approach reflects our concern with maintaining the virulence of *Paracoccidioides brasiliensis* for experimental accuracy. This study was approved by the Ethics Committee on Animal Use (CEUA-UNIFESP) of the Federal University of São Paulo, Brazil, under protocol number CEP 8888301117, (approval date: 2 March 2021). Unless otherwise mentioned, all chemicals were purchased from Sigma-Aldrich (St. Louis, MO, USA).

### 2.2. Growth Assay of P. brasiliensis in Acid Conditions

Growth curves were generated by evaluating fungal counts over several days using Trypan blue dye. *P. brasiliensis* yeast cells were cultivated in F12 medium (supplemented with glucose 2%) for 5 days at 37 °C, with biological duplicates for each condition. Yeast cells (1 × 10^4^; viability >90%) were seeded into a 12-well culture plate containing 2 mL of F12 (supplemented with 2% glucose) at pH 6.5 (control) or pH 4.0, with or without 0.008% BSA, and maintained at 37 °C with constant stirring. The pH was monitored throughout the assay period. After 48 h, the number of viable cells was determined every two days for 18 days using a Neubauer chamber, with viable cells counted in triplicate using Trypan blue exclusion dye. The data were plotted on a graph, and growth curves along with statistical analyses were performed using GraphPad Prism software version 7 (San Diego, CA, USA).

### 2.3. Spot Assay

Fungal sensitivity to stressors was investigated using a spot assay [12]. *P. brasiliensis* (1 × 10^7^) was cultured in mYPD medium at pH 6.5 (control) or pH 4.0 for 0, 24, and 48 h at 37 °C. Serial dilutions of each sample were carried out in mYPD, and 7 µL of each suspension was applied to mYPD agar supplemented with various stress agents: Congo Red (5 µg/mL), Calcofluor White (3 µg/mL), NaCl (100 mM), Menadione (5 µM), or Pepstatin

A (2.5 µM) (an inhibitor of aspartic protease). The plates were incubated for 7 days at 37 °C and then photographed. This assay was performed in biological triplicates.

### 2.4. Evaluation of P. brasiliensis Pigmentation and Growth at Acidic pH

Dry weight and visual observation were used to assess the growth rate of *P. brasiliensis* mycelia and yeast and the production of pigmentation in an acidic environment. *P. brasiliensis* yeast cells were grown on mYPD agar medium at pH 6.5 at 37 °C for 5 days. Then, yeast cells were inoculated in liquid mYPD medium and incubated at 25 °C under 110–150 rpm agitation for 7 days (to reverse yeast to the mycelium phase). After the change, the cells were centrifuged at 1680× *g* for 5 min at room temperature, washed once in PBS buffer, and then mycelium was grown in mYPD medium at pH 6.5 or pH 4.0 at 25 °C under 110–150 rpm agitation for 5 days. The cells were centrifuged at 1680× *g* for 5 min at room temperature, washed once in PBS buffer, and then the concentrated mycelial cells were photographed.

To evaluate the pigmentation of *P. brasiliensis* yeast cells, 1 × 10^7^ yeast cells (viability > 90%) were grown in 150 mL of mYPD medium at pH 6.5 or pH 4.0 at 37 °C under constant agitation (150 rpm) for 5 days [10,12]. It is worth noting that the pH of the medium was monitored every day using pH test strips to confirm that no modifications occurred during the experiment period. Then, the material was centrifuged at 3000× *g* for 10 min at room temperature, washed once in PBS buffer, and then centrifuged again at 3000× *g* for 10 min at room temperature. The supernatant was discarded, and the pellet was photographed. After image acquisition, the cells were resuspended in 100% methanol for 15 min, centrifuged at 1500× *g* for 10 min, and the supernatant was discarded. The pellet was dried in an oven at 60 °C for 20 min. Finally, the dry mass was weighed.

### 2.5. Quantification of Melanin Production in Liquid Culture

Melanin quantification was performed as previously described by Black et al. [15]. *P. brasiliensis* yeast cells (1 × 10^7^ cells/mL) were cultured in mYPD medium (pH 6.5 or pH 4.0) for 7 days at 37 °C under constant agitation (150 rpm). After incubation, the cultures were centrifuged at 3000 × *g* for 10 min at room temperature. The absorbance of the supernatant was measured at 490 nm using a Thermo Plate NM microplate reader to determine the extracellular melanin content. The remaining cell pellet was washed twice with distilled water and digested in 100 μL of 1 M NaOH containing 10% dimethyl sulfoxide (DMSO) for 1 h at 95 °C. The cell digests were centrifuged, and the absorbance of the supernatant was measured at 490 nm to assess the cell wall-associated melanin content. Extracellular melanin values were normalized to the dry weight of the cell mass. All assays were performed in triplicate.

### 2.6. Evaluation of Laccase Activity

*P. brasiliensis* yeast cells (1 × 10^7^) were cultivated in 150 mL of mYPD at pH 6.5 or pH 4.0, at 37 °C with constant agitation (150 rpm) for 5 days. After cultivation, the cells were centrifuged at 3000× *g* for 20 min at 4 °C and washed with 800 µL of water, followed by a second centrifugation at 3000× *g* for 10 min at 4 °C. This washing step was repeated twice. The cells were then mechanically lysed using glass beads in sodium acetate buffer (0.1 M sodium acetate, pH 4.5), with two 10 min vortex cycles and two 5 min cooling intervals on ice. The lysed cells were centrifuged at 2000× *g* at room temperature for 10 min, and the supernatant was collected and analyzed.

Laccase activity was determined using the ABTS oxidation method by Bourbonnais et al. [16]. The assay mixture contained 0.5 mM ABTS, 0.1 M sodium acetate, pH 4.5, and 100 μg of protein extract. Samples were incubated for 5 min at room temperature and the absorbance was measured at 420 nm using a spectrophotometer against an appropriate blank. Oxidation of ABTS was followed by an absorbance increase at A420 (ε420, 3.6 × 10^4^ M^−1^·cm^−1^). Enzyme activity was expressed in units (U = µmol of ABTS oxidized per minute).

### 2.7. Bioinformatic Analysis

To identify the proteins involved in the melanin signaling pathway in the *P. brasiliensis* (Pb18 isolate) genome, the ABR1 and ABR2 proteins from *Aspergillus niger* (ID An14g05370), *ABL1*, *HPPD*, *HMGX*, and *MAIA* from *Aspergillus fumigatus* (ID Afu2g17600), and *HMGA* and *FAHA* from *Aspergillus nidulans* (ID AN1896) were used as parameters due to their greater similarity to the proteins found in *P. brasiliensis* (Pb18 isolate). Protein domains were found by inputting sequences into the Fungi Data Base (FungiDB https://fungidb.org, accessed on 10 September 2023). Additional searches were performed using the Basic Local Alignment Search Tool (https://blast.ncbi.nlm.nih.gov, accessed on 13 September 2023).

### 2.8. P. brasiliensis RNA Isolation

*P. brasiliensis* yeast cells were grown in mYPD broth for 5 days at 37 °C with continuous shaking at 150 rpm. The cells were washed with PBS and resuspended in medium YPD pH 6.5 or 4 for 24 and 48 h. Fully formed yeast cells were mechanically disrupted by vortexing with glass beads for 5 min in TRIzol reagent (Invitrogen, Carlsbad, CA, USA), following the protocol described by Castilho et al. [10]. Pb18 growing without stimulus was used as a control for all the experiments, and fungal viability was evaluated by Trypan blue staining. Cells in TRIzol were processed as suggested by the manufacturer for the purification of RNA, which was then quantified with a NanoDrop 3300 fluorospectrometer (Thermo Fisher Scientific, Wilmington, DE, USA). The quality of the extracted RNA was verified using 2% agarose gel electrophoresis, and the RNA was stored at –80 °C in H_2_O.

### 2.9. Real-Time Quantitative RT-qPCR

The abundance of *P. brasiliensis* transcripts for different experimental conditions was quantified by RT-qPCR. For complementary DNA (cDNA) synthesis, 500 ng of RNA was initially submitted to the DNase I enzyme (Thermo Fisher Scientific, Waltham, MA, USA) and then to ProtoScript First Strand cDNA Synthesis kit (New England BioLabs, Ipswich, MA, USA), according to the manufacturer’s instructions. To assess gene expression by real-time quantitative PCR, the reaction was performed with SYBR^®^ Green Master Mix (Applied Biosystems, Foster City, CA, USA) according to the manufacturer’s instructions. The endogenous expression genes for α-tubulin (α-TUB) and 18S (ribossonal RNA) were used as housekeeping genes. The samples were prepared in triplicate in a 96-well plate (0.2 mL MicroAmp™ Optical 96-Well Reaction Plate—Applied Biosystems) compatible with the equipment used, and the plate was sealed with an optical adhesive (MicroAmp™ Optical Adhesive Film—Thermo Fisher Scientific, Waltham, MA, USA). The equipment used was the ABI StepOne Plus Real-Time PCR System (Applied Biosystems, Foster City, CA, USA) with the following conditions: 10 min at 95 °C, 40 cycles of 15 s at 95 °C, and 1 min at 60 °C. The dissociation curve included an additional cycle of 15 s at 95 °C, 20 s at 60 °C, and 15 s at 95 °C. The curves of oligonucleotide efficiency were evaluated from a cDNA obtained previously and serially diluted (100, 10, 1, and 0.1 ng/µL). The Ct values of each dilution point were determined and used to make the standard curve, which was then used to calculate the primer efficiency (E = 10^(−1⁄slope)–1^ × 100). The relative expression was determined based on the 2^−ΔΔCt^ method [17]. The sequences used for each gene are listed in Supplementary Table S1.

### 2.10. Analysis of the Modulation of Cell Wall Components

Yeast-like cells (1 × 10^7^) of *P. brasiliensis* with viability (>90%) were cultured in 150 mL of mYPD at pH 6.5 or 4, at 37 °C with agitation of 110–150 rpm for 5 days. The cells were washed three times with chilled PBS (4 °C) by centrifugation at 1680× *g* for 10 min at 4 °C. Yeast-like cells (1 × 10^6^ cells/mL) were used for each marker and for the negative control (absence of the marker). The cells were resuspended in 4% formaldehyde for 30 min at room temperature. Subsequently, the cells were washed twice with chilled PBS (4 °C) by centrifugation at 12,000× *g* for 10 min at 4 °C. The samples were incubated in blocking buffer (PBS, 1% BSA) for 1 h at room temperature. Wheat germ agglutinin (WGA) conjugated with FITC (Sigma-Aldrich, St. Louis, MO, USA) at a concentration of 100 µg/mL in 1 mL of PBS was used to evaluate the surface presentation of chitin (WGA-FITC, 100 mg/mL) [18]. Concanavalin A conjugated with FITC (Sigma-Aldrich, St. Louis, MO, USA) at a concentration of 25 µg/mL in 500 µL of PBS was used for mannan determination. The samples for both markers were incubated for 1 h in the dark with agitation at 800× *g*. They were then centrifuged at 12,000× g for 5 min at 4 °C and washed three times with 1 mL of PBS. At the end of the process, the samples were homogenized in 500 µL of PBS [19,20].

For β-glucan labeling, the blocking buffer was supplemented with rabbit serum (PBS, 1% BSA, and 5% rabbit serum). After blocking, the cells were incubated with 1 µg/mL of Fc-Dectin-1 probe (which binds to β-glucans) for 1 h on ice. This probe corresponds to the human Dectin-1 receptor fused with the Fc portion of mouse IgG1 (Sino Biological, Beijing, China). Subsequently, the cells were incubated with the secondary antibody anti-mouse IgG conjugated with Alexa-488 at a ratio of 1:200 for 45 min on ice. Between each incubation step, the cells were washed three times with blocking buffer (PBS, 1% BSA). At the end of the process, the samples were homogenized in 500 µL of PBS. For flow cytometry analysis, the previously labeled cells were centrifuged and homogenized in 500 µL of PBS. Then, data acquisition was performed on a flow cytometer (BD FACSCalibur^TM^, Becton Dickinson, Franklin Lakes, NJ, USA) using the FL-1 detection channel. Each sample was applied in triplicate experiments, and the experiment was conducted in biological triplicates. A total of 10,000 events were counted, and the quantification graphs were generated based on the median fluorescence intensity (MFI). The obtained data were analyzed using FlowJo software version 10.6.2 (FlowJo, LLC, FlowJo™, Ashland, OR, USA) [19,20].

### 2.11. Phagocytosis Assay

The murine macrophage cell line RAW 264.7 was cultured in RPMI 1640 medium (ThermoFisher, Waltham, MA, USA) supplemented with 10% FBS in a humidified atmosphere with 5% CO_2_ until reaching 90% confluence. Then, the cells were plated in 24-well plates (1 × 10^5^ cells/well). Prior to plating, a circular coverslip with a diameter of 15 mm was added to each well. For infection, macrophages were cultured with *P. brasiliensis* yeast in the exponential phase, suspended in RPMI 1640 medium supplemented with 10% FBS at a ratio of 2 yeast cells to 1 macrophage (Multiplicity of Infection—MOI 2:1), and incubated for 24 h at 37 °C and 5% CO_2_. Prior to that, viable yeast-like cells (viability > 90%) of *P. brasiliensis* (1 × 10^7^) were cultured in 150 mL of mYPD at pH 6.5 or 4 at 37 °C with agitation of 110–150 rpm for 5 days, as described before.

After the interaction, each well was washed twice with sterile PBS, the coverslips were stained with the panoptic dye for hematology (Newprov, Paraná, Brazil), and the supernatant was collected for cytokine measurement. The phagocytic index was determined following the protocol established by Popi et al. [21]. The percentage of infection and the number of internalized fungi were evaluated by light microscopy observation. Three hundred cells were counted per slide, and each experimental group was performed in triplicate. It is worth mentioning that culture supernatants were stored at −80 °C for cytokine measurement using the Inflammation Mouse Cytometric Bead Array (CBA) kit (BD). In this case, the following control groups were included: negative control (without treatment and infection); and positive control (group treated with LPS—10 ng/mL).

To evaluate the survival of the fungus within macrophages, the quantification of colony-forming units (CFU) was performed after 24 h of infection. For this purpose, the wells designated for quantification were incubated with 1 mL of sterile chilled ultrapure water and vigorously homogenized to accelerate the macrophage lysis process and release the phagocytosed yeasts. The supernatant was plated on BHI medium supplemented with 10% FBS, adding 100 μL per plate, and incubated for 7 days at 37 °C. After growth, the colony-forming units were counted. This experiment was performed in biological triplicates.

### 2.12. Cytokine Measurement

From the previously described in vitro phagocytosis assay, culture supernatants from the interaction between macrophages and *P. brasiliensis* yeast, cultivated in mYPD at pH 6.5 or pH 4.0 at 37 °C with agitation of 110–150 rpm for 5 days, were collected. The cytokines in the macrophage culture supernatant were evaluated using the Cytometric Bead Array (CBA) method (Inflammation Mouse Kit CBA, BD) following the manufacturer’s instructions (BD Bioscience, San Jose, CA, USA). Sample readings were performed using the FACSCalibur flow cytometer (BD). Cytokine analysis was performed by calculating the standard curve for each cytokine and subsequently analyzing the pg/mL value of each cytokine in each sample. All analyses were performed using the FCAPArray software version 3.0 (BD).

### 2.13. Nitric Oxide (NO) Measurement

Nitric oxide (NO) levels were quantified using the Griess colorimetric assay, which detects nitrite (NO_2_^−^), a stable product of NO oxidation, in culture supernatants previously collected. In triplicate, 100 µL of culture supernatant was transferred to a 96-well half-area plate (Costar), followed by the addition of 100 µL of Griess reagent. The Griess reagent was prepared with 1% sulfanilamide in 2.5% phosphoric acid (H_3_PO_4_) and 0.1% N-1-naphthylethylenediamine dihydrochloride. A standard curve was generated using a sodium nitrite solution initially prepared at 100 µM and serially diluted 2-fold in distilled water. After a 10 min incubation in the dark at room temperature, absorbance was measured at 540 nm using a microplate reader.

## 3. Results

### 3.1. Growth and Adaptation to Stress of P. brasiliensis in Acidic pH

**Figure 1:**
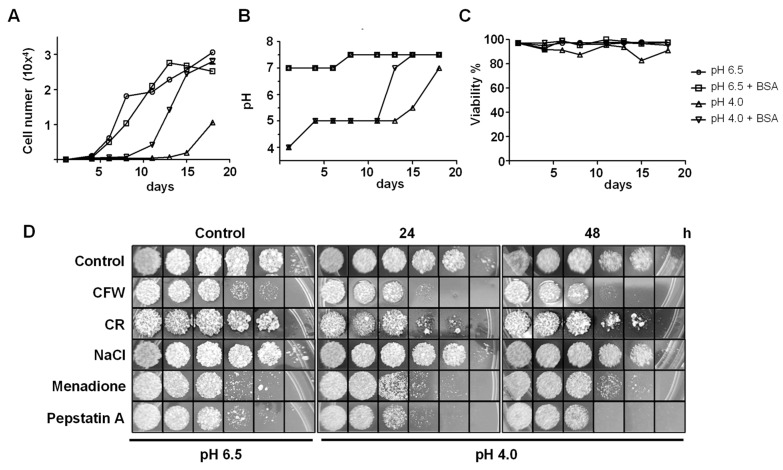
Growth profile, viability, and extracellular pH variation of *P. brasiliensis* cultured under acidic conditions. (**A**) Growth curve of *P. brasiliensis* (Pb18 isolate) in defined F12 medium (2% glucose) adjusted to pH 6.5 or pH 4.0, in the presence or absence of BSA (0.008%), incubated at 37 °C over 18 days. Cultures were initiated with 1 × 10^4^ viable yeast cells. (**B**) Measurement of extracellular pH throughout the incubation period under the same culture conditions. (**C**) Cell viability assessed every 48 h by Trypan Blue exclusion assay. (**D**) Growth profile of yeast cell to stress-inducing agents after growth in mYPD medium at pH 6.5 (control) or pH 4.0 for 24 or 48 h. After incubation, yeast cells (1 × 10^7^ cells/mL) were diluted and plated on solid mYPD medium supplemented with Calcofluor White (CFW, 3 μg/mL), Congo Red (CR, 5 μg/mL), NaCl (100 mM), Menadione (5 µM), and Pepstatin A (2.5 μM). Plates were incubated at 37 °C in a humidified chamber for 7 days. Data shown are representative of three independent experiments performed in triplicate.

To assess the differences between growth stages of *P. brasiliensis*, tests under normal pH conditions (pH 6.5) or acidic pH (pH 4.0) were carried out with the vPb18 isolate (virulent isolate). To assess the growth of vPb18 under acidic conditions (pH 4.0), 1 × 10^4^ cells/mL were seeded in F12 medium (supplemented with glucose) at pH 6.5 (control condition) or pH 4.0, in the presence or absence of 0.008% BSA. Viable cell counting was performed every 48 or 72 h for 18 days. The cells cultured at pH 4.0 showed a 72% growth delay compared to the pH 6.5 control (Figure 1A). No difference in fungus growth was observed between pH 6.5 cultures with or without BSA (Figure 1A). However, cells grown in pH 4.0 supplemented with 0.008% BSA exhibited a 61% growth delay compared to the control (Figure 1A). Fungus growth at pH 4.0 was only observed from the 15th day onward (Figure 1A). This growth coincided with an increase in pH from 5 to 6.5 in the culture medium (Figure 1B). The control cultures (pH 6.5, and pH 6.5 plus BSA) maintained a pH between 7 and 7.5 throughout the evaluated period (Figure 1B). Importantly, cell viability remained above 90% under all evaluated conditions throughout the assay period (Figure 1C). These data demonstrate that *P. brasiliensis* cultivated at pH 4.0 exhibits reduced growth; while maintaining its viability, it can only grow and multiply slowly when the pH of the medium is modulated.

To evaluate the impact of acidic pH on the fungal stress response, *P. brasiliensis* (Pb18) yeast cells were cultured in F12 medium adjusted to pH 6.5 or pH 4.0 for 24 and 48 h at 37 °C. After each incubation period, fungal susceptibility to various stress conditions was assessed (Figure 1D). Yeasts cells grown at pH 6.5 showed reduced growth when exposed to Calcofluor White (CFW, a cell wall-perturbing agent), menadione (an oxidative stress inducer), and pepstatin A (an aspartic protease inhibitor), compared to the non-stressed control (Figure 1D, left panel). No growth differences were observed in the presence of Congo Red (CR, cell wall-perturbing agent) or NaCl (osmotic stress), under the same pH 6.5 condition (Figure 1D, left panel). At pH 4.0, the growth profile after 24 and 48 h remained similar to that observed at pH 6.5 for most of the stressors tested. However, exposure to CR resulted in reduced growth, indicating that sensitivity to this specific cell wall stressor is increased under acidic conditions (Figure 1D, central and right panels). Together, these findings suggest that low external pH does not broadly impair the stress response of *P. brasiliensis*, but selectively affects fungal viability growth in media supplemented with CR. This implies that acidic conditions may compromise cell wall stability, making the fungus more vulnerable to agents that target cell wall integrity.

### 3.2. Low pH Promotes Melanin-like Pigment Formation and Enhances Laccase Activity in P. brasiliensis

**Figure 2:**
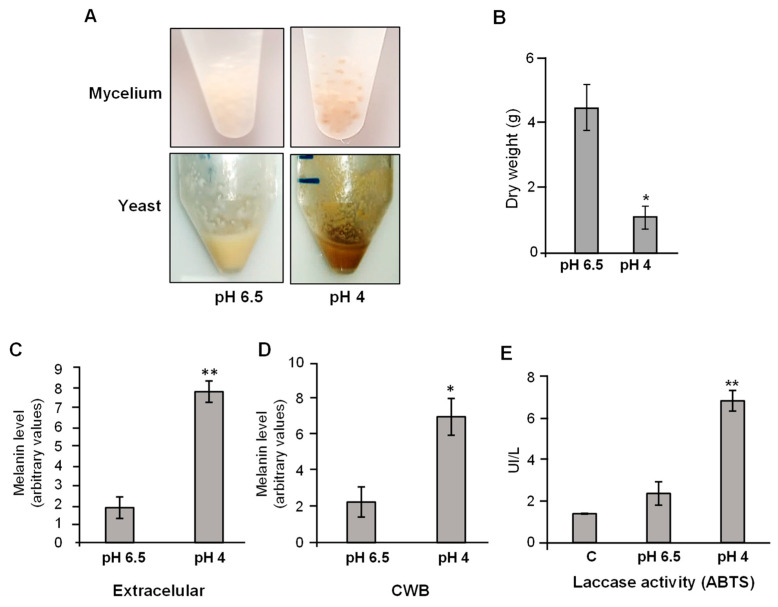
Pigmentation profile and laccase activity of *P. brasiliensis* cultured under acidic conditions. Mycelial and yeast-phase *P. brasiliensis* cells were harvested during the exponential growth phase and incubated in mYPD broth adjusted to pH 6.5 or pH 4.0. Mycelial cultures were maintained at 25 °C, and yeast cultures at 37 °C for 96 h. (**A**) Representative images of mycelial (upper panel) and yeast-phase (lower panel) cultures after incubation under the indicated pH conditions. Cultures were centrifuged at 14,000 × *g* for 15 min at 4 °C, supernatants were discarded, and the resulting cell pellets were photographed. (**B**) Dry weight of yeast cells cultivated at pH 6.5 or pH 4.0. (**C**,**D**) Quantification of extracellular (**C**) and cell wall-bound (CWB) (**D**) melanin by measuring absorbance at OD_490_ (see Methods), normalized to the dry weight of fungal biomass. (**E**) Laccase activity in yeast cells cultured at each pH, assessed using the ABTS oxidation assay and normalized to the dry mass (g). Data represent the mean ± standard deviation of three independent experiments, each performed in triplicate. Statistical significance: * *p* ≤ 0.05; ** *p* ≤ 0.01.

Mycelial and yeast-form cells of *P. brasiliensis* were cultured in mYPD broth, adjusted to pH 6.5 or pH 4.0, for 96 h at 25 °C and 37 °C, respectively. Interestingly, pigment production was observed in both morphotypes when cultured under acidic conditions (Figure 2A). Notably, the yeast-form cell pellet grown at pH 4.0 exhibited a more intense brown coloration compared to the beige hue observed in the control condition (pH 6.5) (Figure 2A, bottom panel). As previously reported, the growth of yeast-form cells was reduced under acidic conditions, as evidenced by dry mass measurements (Figure 2B). Cells cultivated at pH 4.0 displayed a four-fold decrease in biomass compared to the control. Despite the reduced growth, viability remained above 94% in cells exposed to acidic pH. No morphological alterations were observed under bright-field microscopy at 40× magnification.

Melanin quantification after 48 h of incubation showed that cultures grown at pH 4.0 produced higher levels of both extracellular and cell wall-associated melanin compared to those grown at pH 6.5 (Figure 2C). These findings indicate that acidic conditions stimulate pigment production in *P. brasiliensis*. To confirm whether the pigment produced under acidic conditions was melanin-like, we evaluated laccase activity, an enzyme involved in fungal melanin biosynthesis and commonly used as an indirect indicator of melanin production [22]. Laccase activity was measured using the ABTS oxidation assay [16], and yeast cells cultured at pH 4.0 exhibited a four-fold increase in laccase activity compared to those grown at pH 6.5 (control) (Figure 2D). Together, these results suggest that extracellular acidification enhances laccase activity and promotes the synthesis of melanin-like pigments in *P. brasiliensis*.

### 3.3. Acidic pH Induces the Expression of DHN-Melanin Pathway Genes in P. brasiliensis

To determine whether genes involved in melanization contribute to the pigment formation observed in *P. brasiliensis* yeast cells, we first identified genes associated with two known melanin biosynthesis pathways, including (i) the 1,8-dihydroxynaphthalene (DHN)-melanin pathway [23], and (ii) the pyomelanin synthesis pathway via L-tyrosine/L-phenylalanine degradation, which leads to dopaquinone production from L-tyrosine [24]. Gene identification was based on homology with well-characterized pathways in other fungal species [25,26,27]. For the DHN-melanin pathway, the genes *ABR1*, *ABR2*, *ABL1* were identified in *P. brasiliensis* isolate Pb18 based on similarity with orthologs from *Aspergillus niger*, *Aspergillus fumigatus*, and *Aspergillus nidulans*. These genes exhibited at least 25% sequence similarity, indicating conserved homology (Table 1). For the pyomelanin pathway, the genes *HPPD*, *HMGX*, *MAIA*, *HMGA*, and *FAHA*, involved in the degradation of L-tyrosine/L-phenylalanine, showed sequence similarity above 40%, reaching up to 80% in some cases, suggesting that this signaling pathway is highly conserved across fungal species (Table 1).

**Figure 3:**
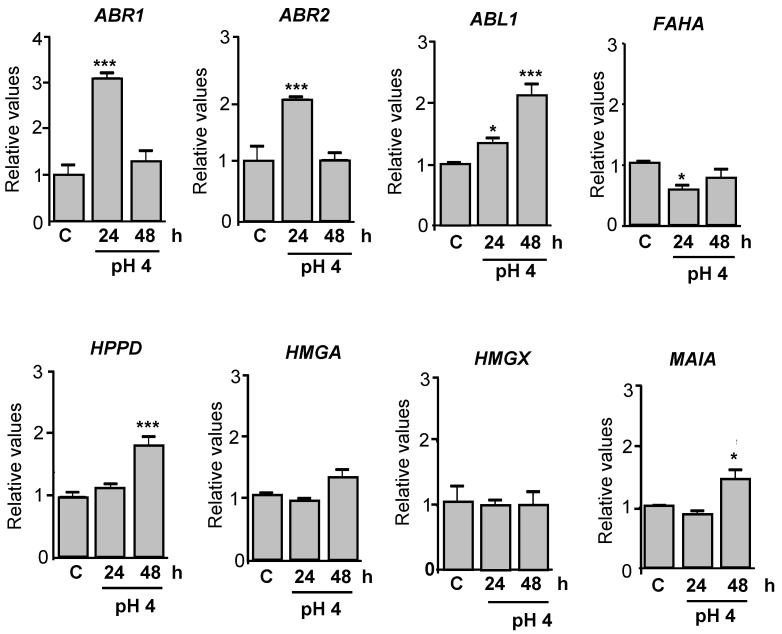
Quantitative PCR analysis of melanin-related genes in *P. brasiliensis* yeast cells cultured under acidic conditions. *P. brasiliensis* yeast cells were cultured in F12 medium supplemented with 2% glucose at pH 6.5 (control) or pH 4.0, for 24 and 48 h. Total RNA was extracted using the TRIzol^®^ method. Quantitative PCR was performed to evaluate the expression of genes involved in the 1,8-dihydroxynaphthalene (DHN) melanin biosynthesis pathway, including *ABR1* (PADG_05994), *ABR2* (PADG_03184), and *ABL1* (PADG_02849), as well as genes associated with the pyomelanin degradation pathway of L-tyrosine/L-phenylalanine: *FAHA* (PADG_08465), *HPPD* (PADG_08468), *HMGA* (PADG_08466), *HMGX* (PADG_08467), and *MAIA* (PADG_08464). Gene expression was normalized to the reference genes *α-TUB* and *18S rRNA*. Data represent the mean ± standard deviation from three independent experiments, each performed in triplicate. * *p* ≤ 0.05, and *** *p* ≤ 0.001.

Based on the identified genes, specific primers were designed, and RT-qPCR analysis was performed using cDNA synthesized from *P. brasiliensis* yeast cells cultured at pH 6.5 (control) or pH 4.0, for 24 and 48 h. Among the genes involved in the DHN-melanin pathway, *ABR1* and *ABR2* exhibited a significant 2- to 3-fold increase in expression after 24 h at pH 4.0, but their expression levels returned to baseline (similar to the control) after 48 h (Figure 3). In contrast, *ABL1* expression was significantly upregulated only after 48 h of acidic cultivation (Figure 3). Regarding the pyomelanin pathway, the *FAHA* gene showed a significant downregulation compared to the control. Conversely, *HPPD* and *MAIA* were significantly upregulated after 48 h at pH 4.0. No changes in expression were observed for *HMGA* and *HMGX* under the tested conditions (Figure 3). These results suggest that the DHN-melanin pathway is likely activated in response to acidic pH and may play a key role in the melanization process of *P. brasiliensis* under environmental stress.

### 3.4. Acidic pH Modulates Cell Wall of P. brasiliensis

**Figure 4:**
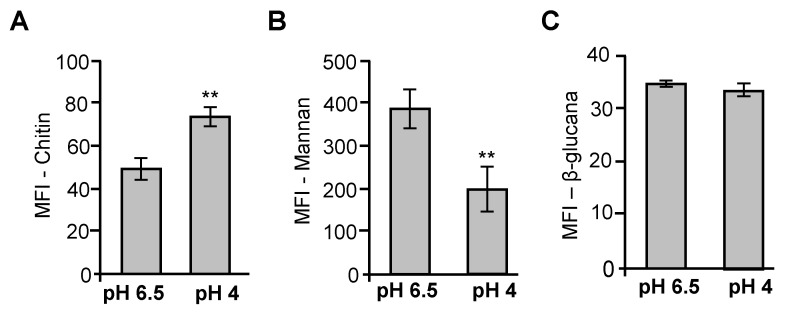
Analyses of *P. brasiliensis* cell wall components after cultivation under acidic pH conditions. Yeast cells of *P. brasiliensis* were cultured at pH 6.5 or pH 4.0 for 96 h and subsequently labeled with wheat germ agglutinin (WGA) conjugated to FITC for chitin oligomers (to detect chitin on the surface) (**A**), and Concanavalin A conjugated to FITC for mannans (**B**) and Fc-Dectin-1 conjugated to Alexa Fluor 488 for β-(1,3)-glucan detection (**C**). Quantification of cell wall components based on median fluorescence intensity (MFI). Error bars represent the standard deviation of triplicate measurements. ** *p* ≤ 0.01.

The fungal cell wall is crucial for preserving cell morphology and providing resistance to external stresses encountered during host colonization. The use of Congo Red (CR) in growth media serves as an experimental approach to disrupt the cell wall, as the dye binds to crystalline polymers such as chitin and cellulose [28]. As previously observed, the cell wall-perturbing agent CR reduced the viability of yeast cells cultured under acidic pH conditions (Figure 1D). Therefore, we opted to evaluate the modulation of the main components of the fungal cell wall after cultivation at acidic pH. In this assay, we evaluated mannan and β-glucan levels, and chitin detection on the cell wall surface of *P. brasiliensis* yeast cells cultivated at pH 4.0 for 96 h. Cells cultivated at pH 4.0 for 96 h exhibited a significant increase in exposed chitin levels (Figure 4A) and a decrease in mannan content (Figure 4B), when compared to the control condition (pH 6.5). However, no changes were observed in β-glucan levels (Figure 4C). Representative histograms of the flow cytometry analysis for each labeled cell wall component are shown in Supplementary Figure S1. These data demonstrate that cell wall components are modulated in cells cultivated in an acidic environment.

### 3.5. Growth Under Acidic Conditions Decreases Phagocytosis of P. brasiliensis and Alters Cytokine and Nitric Oxide Production by Macrophages

**Figure 5:**
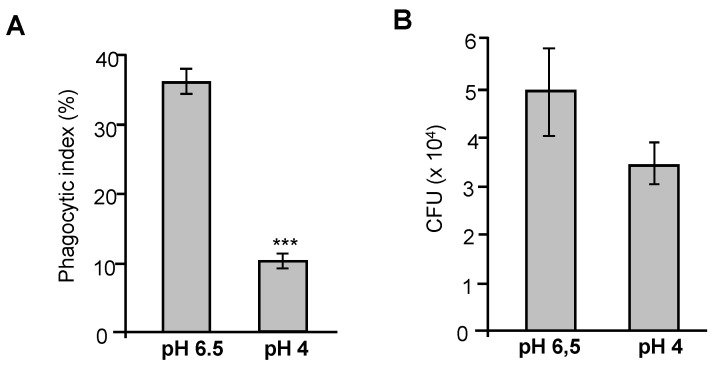
Evaluation of the phagocytic index of *P. brasiliensis* yeasts cultured under acidic conditions. Murine RAW 264.7 macrophages (2.5 × 10^5^ cells) were seeded in RPMI medium supplemented with 10% FBS. *P. brasiliensis* yeast cells were previously cultured for 96 h in YPDm medium at pH 6.5 (control) or pH 4.0. The interaction assay was performed at a ratio of 2:1 (yeasts/macrophages) for 24 h at 37 °C with 5% CO_2_. After incubation, the cells were stained and observed under a light microscope for quantification of internalized yeasts. (**A**) The phagocytic index was determined by counting 300 macrophages across different microscopic fields. (**B**) Colony-forming units (CFUs) of yeast cells recovered from macrophages after interaction. *** *p* ≤ 0.001.

Our results indicated that exposure to acidic pH modulates the composition of the *P. brasiliensis* cell wall and induces melanin production. To assess whether these changes affect macrophage response, particularly those of RAW 264.7 cells, key innate immune phagocytes involved in *P. brasiliensis* recognition and clearance, we evaluated the phagocytic index and survival of yeast cells cultured under different pH conditions. Macrophages were incubated with yeasts cells (1:2 ratio) previously cultured at pH 6.5 (control) or pH 4.0. After 24 h of co-incubation, the phagocytosis index and the number of colony-forming units (CFUs) representing viable intracellular fungi were assessed. Yeast cells cultured at pH 4.0 exhibited a 3.5-fold reduction in the phagocytic index compared to control cells (Figure 5A). Despite this, no statistical difference in CFU counts was observed between the two conditions (Figure 5B), although a slight reduction in CFUs was noted in the pH 4.0 group. This difference likely reflects the reduced number of yeast cells internalized by macrophages under acidic conditions (Figure 5A). Representative images of macrophages containing phagocytosed *P. brasiliensis* yeast cells are shown in Supplementary Figure S2.

**Figure 6:**
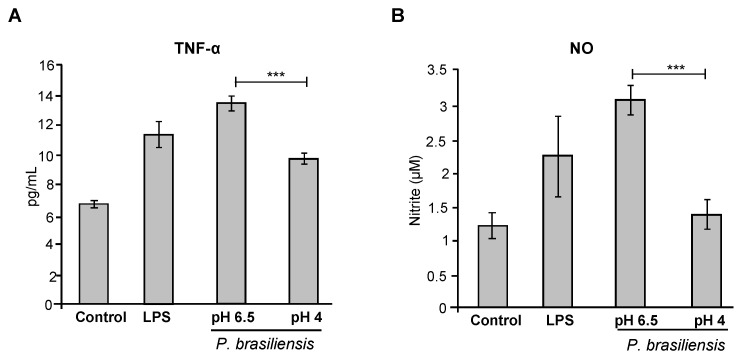
Quantification of TNF-α and nitric oxide (NO) in the supernatant of RAW 264.7 cells after interaction with *P. brasiliensis* cultured at acidic pH. (**A**) TNF-α levels in the supernatant of RAW 264.7 macrophages following interaction with *P. brasiliensis* cultured at pH 6.5 (control) or pH 4.0. Negative control: macrophages not incubated with *P. brasiliensis*; positive control: macrophages stimulated with LPS (100 ng/mL). (**B**) Nitric oxide (NO) levels in the supernatant of RAW 264.7 macrophages after interaction with *P. brasiliensis* cultured at pH 6.5 or pH 4.0. *** *p* ≤ 0.001.

The cytokine profiles in the supernatant of macrophage cultures incubated with *P. brasiliensis* yeast cells grown at pH 6.5 (control) or pH 4.0 were evaluated. Cytokine quantification was performed using the Mouse Inflammation CBA kit (BD Biosciences), which allows the quantification of the following cytokines: Interleukin-6 (IL-6), Interleukin-10 (IL-10), Monocyte Chemoattractant Protein-1 (MCP-1), Tumor Necrosis Factor (TNF), and Interleukin-12p70 (IL-12p70). Culture supernatants collected after 24 h of co-incubation were analyzed, and macrophages stimulated with lipopolysaccharide (LPS) were used as a positive control. Among all the cytokines analyzed, only TNF-α was detected at measurable levels. As shown in Figure 6A, macrophages incubated with yeast cells grown at pH 6.5 produced significantly higher levels of TNF-α compared to the unstimulated control. The magnitude of this response was comparable to that induced by LPS. In contrast, macrophages incubated with yeast cells cultured at pH 4.0 produced significantly lower levels of TNF-α compared to those stimulated with control (pH 6.5) yeasts (Figure 6A). A similar profile was observed for nitric oxide (NO) production. Macrophages challenged with yeast cells grown at pH 6.5 secreted elevated NO levels compared to the unstimulated control (Figure 6B). However, NO production was markedly reduced in macrophage cultures exposed to yeast cells grown at pH 4.0, reaching levels similar to those of the negative control (unstimulated macrophages) (Figure 6B). Taken together, these data suggest that the *P. brasiliensis* cells adapted to acidic pH not only exhibit reduced phagocytosis by macrophages but also attenuate the macrophage pro-inflammatory response, including TNF-α and NO production.

## 4. Discussion

Our findings revealed that *P. brasiliensis* undergoes a complex and coordinated adaptation in response to acidic extracellular pH (pHex), a condition that the pathogen likely encounters in vivo within host phagolysosomes, inflamed tissues, or granulomatous lesions [29,30,31,32,33]. In our model, exposure to pH 4.0 led to a substantial delay in fungal growth, yet cell viability remained above 90% throughout the culture period (Figure 1A–C), indicating that the fungus activates survival mechanisms under acidic conditions. Notably, resumption of growth was only observed after alkalinization of the medium, suggesting that *P. brasiliensis* actively modulates the pHex to promote its proliferation.

This extracellular alkalinization may be driven by the secretion of ammonia, a byproduct of amino acid metabolism that has been implicated in pH regulation and virulence in other fungal pathogens [34,35,36,37,38]. Interestingly, the addition of BSA, a nitrogen source, accelerated the recovery of growth and the shift in pHex, reinforcing the hypothesis that nitrogen metabolism and ammonia release are involved in this adaptive response.

Acid stress also triggered changes in fungal susceptibility to cell wall stressors. Yeast cells cultured at pH 4.0 displayed decreased viability after treatment with CR but not to CFW or other stress agents, indicating selective alterations in the fungal cell wall (Figure 1D). This observation was further supported by flow cytometry analysis, which revealed an increase in exposed chitin and a decrease in mannan content in the cell wall, with no significant change in β-glucan levels (Figure 4A–C). Yeasts respond to changes in acidic pHex by remodeling the cell wall [39]. Such remodeling could be a consequence of acid-induced activation of cell wall biosynthetic or repair pathways, as seen in other fungi where acid stress induces the expression of genes related to cell wall integrity (CWI) and to general stress response (GSR) [40,41,42].

These cell wall changes may contribute to immune evasion. Mannans are potent pathogen-associated molecular patterns (PAMPs) recognized by macrophage receptors such as the mannose receptor and TLR4 [43]; thus, their reduction could mask the fungus from host recognition. Simultaneously, increased chitin exposure may alter immune signaling. Chitin has been shown to exhibit both pro- and anti-inflammatory effects depending on host context [18,44,45]. The increased chitin exposure on the cell wall surface of *P. brasiliensis* under acidic conditions could contribute to reduced immune activation.

Indeed, these structural changes had functional consequences in our macrophage interaction assays. Yeasts cultured at pH 4.0 were less efficiently phagocytosed by RAW 264.7 macrophages (Figure 5A) and induced lower levels of TNF-α and nitric oxide (NO) compared to control cells cultured at neutral pH (Figure 6A–B). These data suggest that acid-adapted *P. brasiliensis* is less immunostimulatory and more resistant to macrophage-mediated clearance, possibly due to the combined effects of cell wall remodeling and pigment production.

In parallel, we observed that acidic pH strongly induced the production of melanin-like pigments in both yeast and mycelial phases of *P. brasiliensis*, as evidenced by visible pigmentation and increased quantification of both extracellular and cell wall-associated melanin (Figure 2A–C). Melanin biosynthesis was supported by a significant increase in laccase activity, a key enzyme in pigment formation (Figure 2D). Moreover, RT-qPCR analysis demonstrated the upregulation of genes involved in both DHN-melanin biosynthesis (e.g., *ABR1*, *ABR2*, *ABL1*) and pyomelanin degradation pathways (e.g., *HPPD*, *MAIA*) (Figure 3), indicating that melanin production under acid stress is transcriptionally regulated and likely involves multiple biosynthetic routes.

Melanin is a well-known fungal virulence factor associated with resistance to phagocytosis, oxidative stress, and antimicrobial peptides [7,46,47]. In *P. brasiliensis*, melanized yeast cells have been previously shown to survive better in infected tissues and resist clearance by alveolar macrophages [7,46]. Our data are consistent with these findings and extend them by showing that acidic pH is sufficient to induce melanization in *P. brasiliensis*, even in the absence of exogenous inducers such as L-DOPA or copper [48,49]. This suggests that environmental pH may play a direct role in regulating virulence-associated traits in this fungus.

Taken together, our results indicate that acidic pH triggers a multifaceted adaptation in *P. brasiliensis*, including (i) regulation of extracellular pH via nitrogen metabolism, (ii) remodeling of the fungal cell wall, (iii) stimulation of melanin production, and (iv) reduced recognition and response by macrophages. These processes may contribute to the pathogen’s survival in hostile environments, particularly within the host. Future studies should explore the regulatory networks underlying these adaptations, including the specific roles of the CWI and GSR pathways, ammonia production, and the epigenetic modulation of stress responses.

## 5. Conclusions

The present study demonstrates that acidic pH modulates the physiology and virulence-related traits of *P. brasiliensis*. Under low-pH conditions, fungal growth was delayed, yet viability remained high, with eventual adaptation through medium alkalinization. Acidic pH induced melanin-like pigment production via upregulation of the DHN-melanin pathway and increased laccase activity, while also altering cell wall composition (increased chitin exposure on the cell wall surface, reduced mannan). These modifications reduced macrophage phagocytosis and dampened pro-inflammatory responses (lower TNF-α and NO production), suggesting a potential immune evasion mechanism. Together, these findings highlight how acidic environments may enhance *P. brasiliensis* pathogenicity by promoting stress resistance, melanization, and immune modulation, providing new insights into its adaptive strategies during pathogenesis.

## Figures and Tables

**Table 1:** Characteristics of the genes involved in melanin synthesis in *P. brasiliensis*.

Gene	Organism	ID	ID *P. brasiliensis*	Similarity (%)
*ABR1*	*A. niger*	An14g05370	PADG_05994	29.67
*ABR2*	*A. niger*	An01g13660	PADG_03184	44.25
*ABL1*	*A. fumigatus*	Afu2g17600	PADG_02849	25.88
*HPPD*	*A. fumigatus*	Afu2g04200	PADG_08468	80.5
*HMGX*	*A. fumigatus*	Afu2g04210	PADG_08467	41.18
*MAIA*	*A. fumigatus*	Afu2g04240	PADG_08464	55.75
*HMGA*	*A. nidulans*	AN1897	PADG_08466	78.57
*FAHA*	*A. nidulans*	AN1896	PADG_08465	72.47

## Data Availability

The original contributions presented in this study are included in the article/Supplementary Material. Further inquiries can be directed to the corresponding author.

## Supplementary Materials

The following supporting information can be downloaded at: https://www.mdpi.com/article/10.3390/jof11070504/s1. Figure S1: Representative histograms of the analysis of the labeling of each cell wall component by flow cytometry; Figure S2: Representative images of the phagocytosis assay of *P. brasiliensis* grown at pH 6.5 or pH 4.0; Table S1: Oligonucleotides used for real-time quantitative PCR analysis.
