# Supplementary material for: Acidic pH Modulates Cell Wall and Melanization in Paracoccidioides brasiliensis, Affecting Macrophage Interaction
Source: J Fungi (Basel). 2025 Jul 4;11(7):504. doi: 10.3390/jof11070504 (PMC12296031; doi:10.3390/jof11070504)
Supplement: Supplementary file 1 [file jof-11-00504-s001.zip › Figures S1 and S2.pdf]

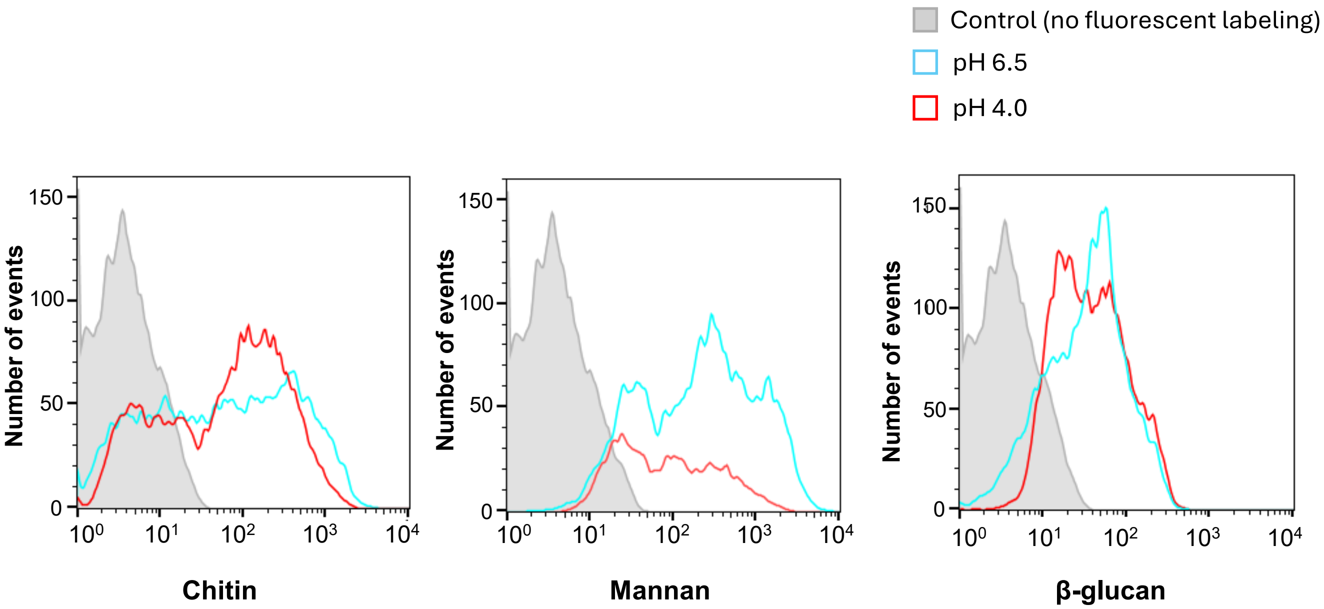

Figure S1. Representative histograms of cell wall component labeling by flow cytometry. Yeast cells of *P. brasiliensis* were cultured at pH 6.5 or pH 4.0 for 96 h, followed by labeling with specific probes: WGA-FITC for chitin oligomers, Concanavalin A-FITC for mannans, and Fc-Dectin-1-Alexa 488 for β-(1,3)-glucans. The negative control corresponds to unstained cells (no fluorescent labeling). Histograms illustrate the fluorescence intensity corresponding to each cell wall component.

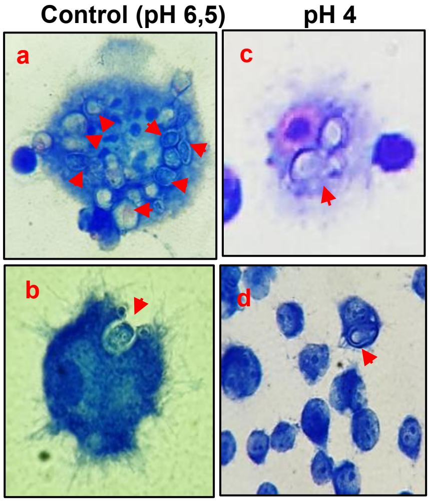

Figure S2. Representative images of the phagocytosis assay of *P. brasiliensis* cultured at different pH levels. Murine RAW 264.7 macrophages (2.5 × 10^5 cells/well) were seeded in RPMI medium supplemented with 10% FBS. *P. brasiliensis* yeasts were previously cultured for 96 h in YPDm medium adjusted to pH 6.5 (control) or pH 4.0. Macrophages and yeasts were co-incubated at a 2:1 ratio (yeast:macrophage) for 24 h at 37 °C in a 5% CO_2 atmosphere. After incubation, cells were stained and examined by light microscopy to assess yeast internalization. Panels (a–b) show macrophages interacting with yeasts cultured at pH 6.5; panels (c–d) show interaction with yeasts cultured at pH 4.0.
