# Supplementary material for: Acidic pH Modulates Cell Wall and Melanization in Paracoccidioides brasiliensis, Affecting Macrophage Interaction
Source: J Fungi (Basel). 2025 Jul 4;11(7):504. doi: 10.3390/jof11070504 (PMC12296031; doi:10.3390/jof11070504)
Supplement: Supplementary file 1 [file jof-11-00504-s001.zip › Table S1.pdf]

Table S1. Oligonucleotides used for real-time quantitative PCR analysis.

Gene		Sequence (5′→3)
ABR1	**Sense**	CCGTCCCGTTGATTGTCTT
	**Anti Sense**	CCGCAAGAGACCCAGTATTT
ABR2	**Sense**	CATCTCACTGCGGCAGAATA
	**Anti Sense**	GACCGATCCCTTCCCATTTATC
ABL1	**Sense**	ATTGGGAGAGCCCTGATTATTT
	**Anti Sense**	CTCCAAGCATCCGACGATATT
HPPD	**Sense**	GATTGGGAGGAGATGGAGAAAG
	**Anti Sense**	GCGGAGAATTCAGTGCAGATA
HMGX	**Sense**	TGCTCCGACTCCACAGATA
	**Anti Sense**	CCAAGCTGGAAATCCCATACT
MAIA	**Sense**	CTGCGAAGGAGGATACCAATAC
	**Anti Sense**	TGGATTGGGTGATTGGGAAG
HMGA	**Sense**	CTTGGCTCTACCGCATCTTAC
	**Anti Sense**	CTGCTTGATCGAGTGGGTATC
FAHA	**Sense**	CCCTTACCCTGAACTACTCAAATC
	**Anti Sense**	CGGCGTAGAAGTCTGTGTAATC
α-TUB	**Sense**	GTGGACCAGGTGATCGATGT
	**Anti Sense**	ACCCTGGAGGCAGTCACA
*18S**	**Sense**	CGGAGAGAGGGAGCCTGAGAA
	**Anti Sense**	GGGATTGGGTAATTTGCGC
